# Post-Transcriptional Control of the *Escherichia coli* PhoQ-PhoP Two-Component System by Multiple sRNAs Involves a Novel Pairing Region of GcvB

**DOI:** 10.1371/journal.pgen.1003156

**Published:** 2013-01-03

**Authors:** Audrey Coornaert, Claude Chiaruttini, Mathias Springer, Maude Guillier

**Affiliations:** UPR9073 du CNRS, Université Paris Diderot, Sorbonne Paris Cité, Institut de Biologie Physico-Chimique, Paris, France; Universidad de Sevilla, Spain

## Abstract

PhoQ/PhoP is a central two-component system involved in magnesium homeostasis, pathogenicity, cell envelope composition, and acid resistance in several bacterial species. The small RNA GcvB is identified here as a novel direct regulator of the synthesis of PhoQ/PhoP in *Escherichia coli*, and this control relies on a novel pairing region of GcvB. After MicA, this is the second Hfq-dependent small RNA that represses expression of the *phoPQ* operon. Both MicA and GcvB bind *phoPQ* mRNA *in vivo* and *in vitro* around the translation initiation region of *phoP*. Binding of either small RNA is sufficient to inhibit ribosome binding and induce mRNA degradation. Surprisingly, however, MicA and GcvB have different effects on the levels of the PhoP protein and therefore on the expression of the PhoP regulon. These results highlight the complex connections between small RNAs and transcriptional regulation networks in bacteria.

## Author Summary

Regulation of bacterial gene expression participates in the ability of these microorganisms to quickly adapt to their environment. This regulation can occur at every level of gene expression. For instance, two-component systems are involved in transcriptional control, while small RNAs usually act at the post-transcriptional level. In this study, the pleiotropic small RNA GcvB is identified as the second small RNA regulator of the central PhoQ/PhoP two-component system, which highlights the connections between the different types of regulation.

## Introduction

Gene regulation in response to environmental conditions is a key feature of bacterial cells, that allows their development in multiple and diverse niches. While this was originally thought to rely only on transcriptional control by proteins, it is now well established that mechanisms underlying the control of gene expression are much more diverse. For instance, numerous examples of post-transcriptional control have been reported that can be mediated by proteins, riboswitches or trans-acting small RNAs (sRNAs) [1].

Even though the first example of a chromosomally-encoded bacterial sRNA regulating the expression of a target-gene encoded at a different locus was described almost 30 years ago [2], it is only in the last decade that our understanding of the number and the role of sRNAs in bacterial physiology has greatly improved [3]. Among those, the Hfq-dependent sRNAs have been extensively studied. This class of sRNAs imperfectly pairs to target-mRNA(s), which in most cases occludes the ribosome binding site (RBS) of the target-gene and therefore down-regulates its expression through translational inhibition. This is often accompanied by degradation of the target-mRNA, either as a consequence of the translation inhibition and/or independently of this process [4]. It is also known that sRNAs can activate gene expression, again by increasing translation and/or stability of their target(s) through base-pairing interactions [5]–[8].

Hfq both prevents the sRNAs from being degraded, and facilitates and stabilizes sRNA-mRNA duplexes. As a result, a productive interaction between an Hfq-dependent sRNA and its target relies only on short and imperfect duplexes. Most, if not all, Hfq-binding sRNAs have multiple targets and, in parallel, a single target can be regulated by multiple sRNAs. This, in addition to the great number of sRNAs in bacterial species (>80 in *E. coli* for instance), contributes to the importance of these molecules in bacterial physiology. One of the best examples is probably the Hfq-dependent GcvB sRNA that has been shown to target more than 20 different mRNAs, most of them probably directly. Its transcription is activated by the product of the adjacent gene, GcvA, a regulator that also controls the *gcvTHP* operon as well as its own transcription [9]. Whereas GcvA negatively autoregulates its own synthesis, it can either activate or repress expression of *gcvTHP*, the glycine cleavage operon whose products catalyze the oxidation of glycine into carbon dioxide, ammonia and a one carbon-unit that will be transferred to tetrahydrofolate. Whether GcvA activates or represses *gcvTHP* operon expression depends on the presence of the GcvR protein and/or glycine. The GcvA/GcvR complex acts as a repressor; but in the presence of glycine, it is disrupted, allowing GcvA to activate the synthesis of the glycine cleavage system. In contrast, purines seem to promote repression. Similarly, *gcvB* transcription requires GcvA and is repressed by GcvR unless glycine is present. In addition, the Lrp global regulator has a positive effect on *gcvTHP* expression [10], but represses *gcvB* expression [9], [11].

As a result of this control by GcvA, GcvB is mostly present in fast-growing cells in rich medium [12]. It negatively controls expression of multiple targets involved in aminoacid transport and metabolism [9], [12]–[14]. As is often the case for sRNAs with several targets, a unique region of the sRNA, referred to as R1 for GcvB, pairs with almost all targets. This region is very well conserved, single-stranded and GU-rich [12]. So far, only 3 targets have been found to be regulated by GcvB independently of its R1 region: *lrp*, *gdhA* and *cycA* [13].

Also highlighting the importance of sRNAs is the fact that several of them target transcriptional regulators. This is true for instance for the master regulator of stationary phase RpoS, whose expression is post-transcriptionally controlled by at least 4 distinct sRNAs [8], [15]–[17]. Several two-component systems (TCS), such as EnvZ/OmpR or DpiA/DpiB, have also been shown to be repressed by sRNAs [18], [19]. Similarly, we have shown in a previous work that MicA, an RpoE-dependent sRNA known to repress the synthesis of multiple proteins [20], many of which are located in the outer membrane [21]–[23], was a direct regulator of PhoQ/PhoP synthesis [24]. This TCS is a central regulatory system in which the PhoQ sensor protein controls the phosphorylation status of the cognate response regulator PhoP, so that it is activated (i.e. phosphorylated) upon low magnesium conditions or in presence of antimicrobial peptides. Under such conditions, PhoP directly regulates dozens of genes involved in major cellular functions such as magnesium homeostasis, bacterial virulence, cell envelope composition and acid resistance [25]. Our previous findings linked therefore the expression of *phoPQ* operon to cell envelope stress through the regulatory sRNA MicA. In addition, they strongly suggested the existence of at least another Hfq-dependent sRNA controlling expression of *phoPQ* at the post-transcriptional level. In this study, we identify GcvB as such an sRNA, and address the mechanism as well as the physiological consequences of this control on the expression of the PhoP regulon.

## Results

### Identification of GcvB as a Regulator of *phoP* Expression

**Figure 1:**
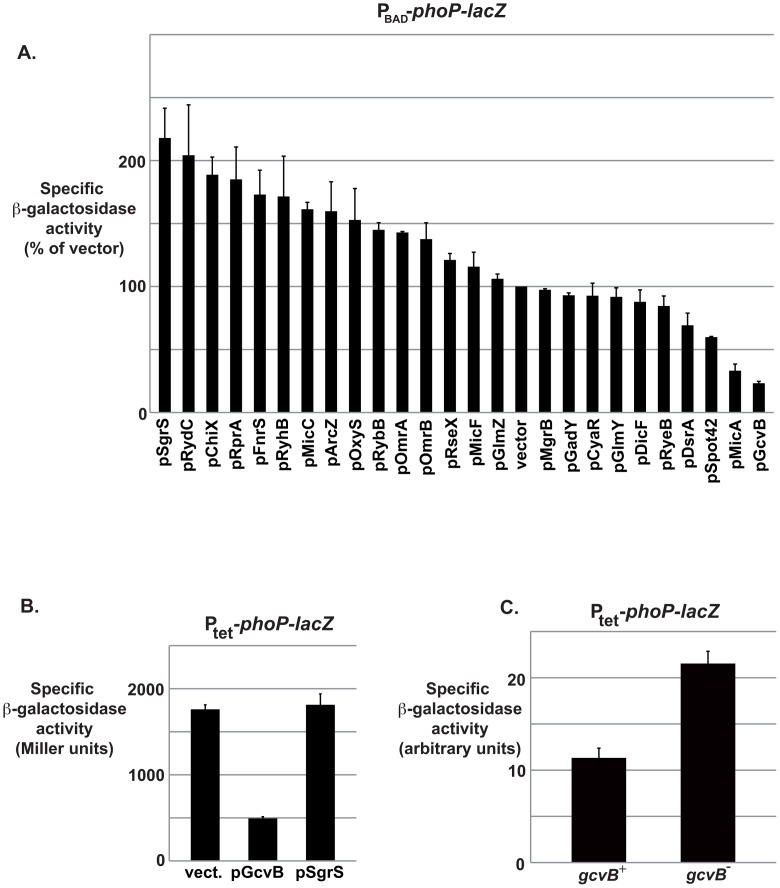
GcvB negatively regulates *phoP* expression at the post-transcriptional level. (A) Specific ß-galactosidase activity of strain MG1425 transformed with an sRNA dedicated-plasmid library. Activity of the strain transformed with the vector control was arbitrarily set at 100%. Plasmid pIS118, overexpressing the IS118 sRNA, was not included in this experiment, but it did not affect expression of P_tet_-*phoP-lacZ* fusion by more than 2-fold (data not shown). (B) Specific ß-galactosidase activity of strain MG1511 transformed with plasmids overexpressing GcvB or SgrS, or with the pBRplac empty vector. (C) Specific ß-galactosidase activity of strains MG1511 (*gcvB^+^*) and MG1521 (*gcvB^−^*).

Even though MicA is an Hfq-dependent sRNA, expression of *phoP* was found to be strongly activated at the post-transcriptional level by the deletion of *hfq* in both wt and *micA* deleted cells [24]. We thus hypothesized that one or several Hfq-dependent regulator(s) could affect *phoP* expression independently of MicA. Therefore, we transformed a strain carrying a P_BAD_-*phoP-lacZ* reporter fusion with a plasmid library overexpressing most of the known *E. coli* Hfq-dependent sRNAs from an IPTG-inducible modified P_lac_ promoter [8]. Transcription of the *phoP-lacZ* fusion is driven by the P_BAD_ promoter so that expression of this fusion should not be sensitive to control of *phoP* at the transcription initiation level. The transcription start site is expected to be identical to that of the proximal *phoP* promoter, P_1_, which is normally positively regulated by PhoP in *E. coli* [26]. The fusion encompasses only 66 nts of *phoP* mRNA, that correspond to a 36 nts 5′ leader followed by the first 30 nts of the ORF. The ß-galactosidase activity of the different transformants was assayed and the results are shown in Figure 1A. Of the 25 sRNAs tested, 4 modulated the expression of the fusion by more than 2-fold, SgrS and RydC positively and MicA and GcvB negatively. Since SgrS is involved in sugar metabolism [27], we suspected that its overproduction could affect expression from the arabinose-induced P_BAD_ promoter. To test this possibility, we measured the SgrS-mediated repression of the same *phoP-lacZ* fusion when constitutively expressed from the P_tet_ promoter instead of P_BAD_. Since the pSgrS plasmid had no effect on this P_tet_-*phoP-lacZ* fusion (Figure 1B), it is likely that it activated the P_BAD_- driven fusion at the promoter level and this was not investigated further. The RydC sRNA has been shown to activate (repress) the expression of fusions that are negatively (positively) regulated by Hfq, most likely by titrating Hfq [28]. One possibility is therefore that it acts on P_BAD_-*phoP-lacZ* in the same way, but further experiments are required for a definitive proof. The same may be true for sRNAs such as ChiX, that also activates the expression of the fusion almost 2-fold.

In the experiment shown in Figure 1A, pMicA repressed the expression of *phoP-lacZ* by 3.1-fold, which is in agreement with our previous results. Furthermore, this experiment also identified GcvB as a multicopy repressor of *phoP-lacZ*, since pGcvB was responsible for a 4.5-fold decrease in the ß-galactosidase activity of the fusion. This last result was confirmed using the P_tet_-*phoP-lacZ* fusion (repression of 3.5-fold, Figure 1B), indicating that, as shown previously for MicA [24], GcvB most likely acts at the post-transcriptional level. Importantly, this repressor effect of GcvB was also visible when GcvB was expressed from the chromosome; a deletion of the *gcvB* gene was sufficient to increase expression of *phoP-lacZ* by 1.9-fold (Figure 1C).

### GcvB and MicA Act Independently on *phoP*

**Figure 2:**
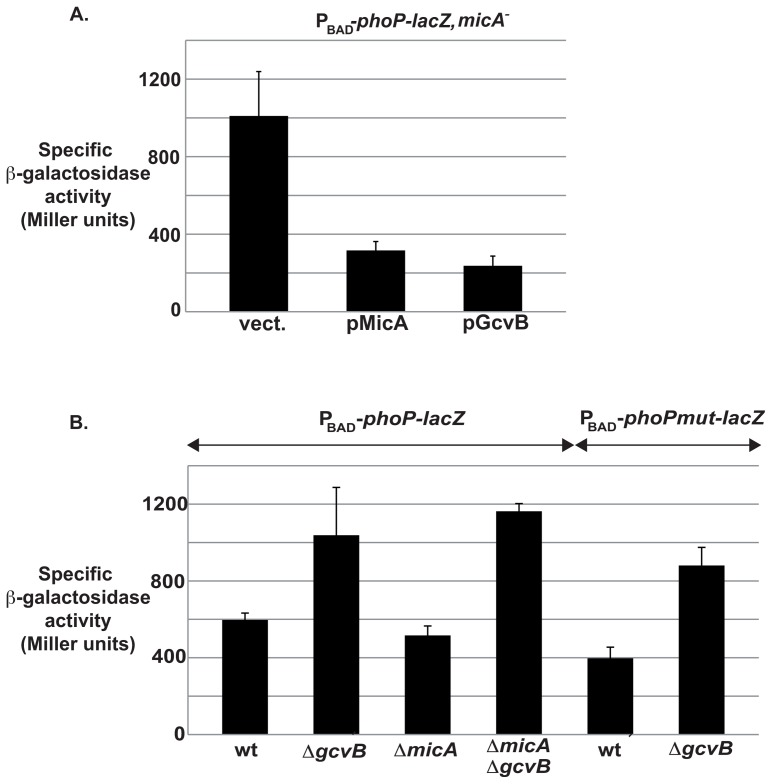
Control of *phoP* by GcvB is independent of MicA. Specific ß-galactosidase activity of the P_BAD_-*phoP-lacZ* fusion was assessed in a *micA* deleted strain (strain MG1452) upon overexpression of MicA or GcvB (A) or in strains deleted for *micA*, *gcvB* or both (B). Strains used in the experiment of panel B are MG1425 (wt), MG1768 (*ΔgcvB*), MG1430 (*ΔmicA*) and MG1709 (*ΔmicA ΔgcvB*) for the wt fusion; and MG1428 (wt) and MG1769 (*ΔgcvB*) for the *phoPmut-lacZ* fusion.

One possible explanation for these results is that GcvB regulates the *phoP-lacZ* fusion by controlling the synthesis and/or activity of a post-transcriptional regulator of *phoP*. Since MicA is so far the only post-transcriptional regulator of *phoP* known to affect our *phoP-lacZ* fusion, we analyzed the effect of GcvB on *phoP* expression in the absence of MicA. In this context, overproduction of MicA and GcvB from a plasmid caused a 3.4- and 4.3-fold decrease respectively in the activity of the *phoP-lacZ* fusion (Figure 2A), which is similar to what was observed in *micA^+^* cells. Consistent with this observation, deletion of *gcvB* resulted in a 1.7- or 2.3-fold activation of *phoP-lacZ* in wt or *micA^−^* cells respectively (Figure 2B). Therefore, GcvB acts independently of MicA to regulate *phoP* expression. In this experiment, deletion of *micA* has no significant effect on the expression of *phoP*, because transcription of MicA is dependent on the RpoE sigma factor, which is not activated under the experimental conditions of Figure 2B.

**Figure 3:**
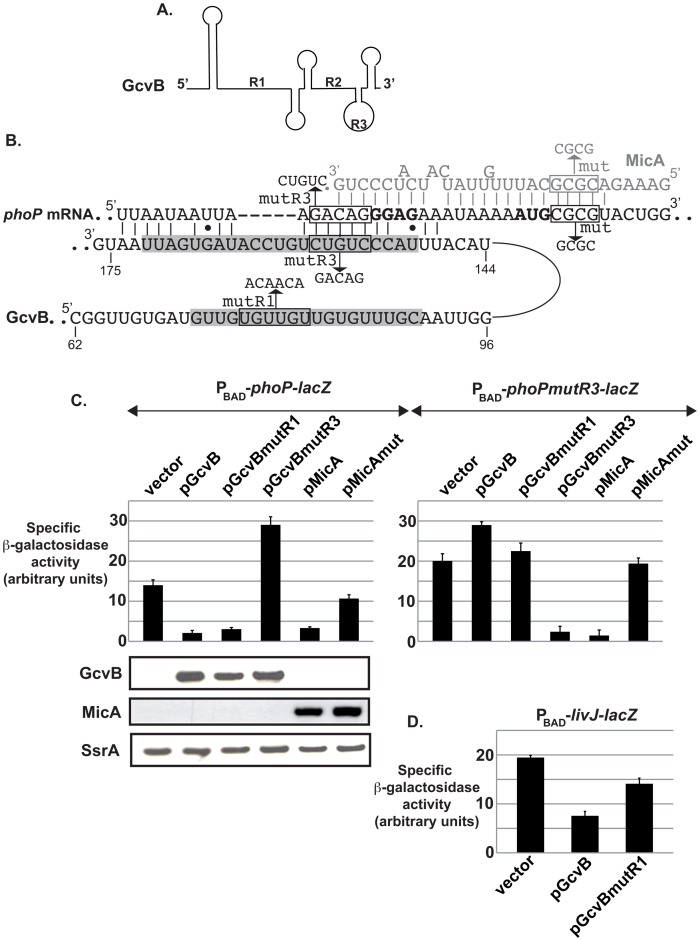
GcvB directly controls *phoP in vivo* through a novel pairing region. (A) Schematic of GcvB secondary structure based on previously published experimental data [12]. (B) Predicted base-pairing interaction between GcvB and *phoPQ* mRNA using the TargetRNA program. Numbering of GcvB nts is relative to the transcription start site. Nature of mutations mut and mutR3 in *phoP* (that abolish control by MicA and GcvB respectively) are indicated as well as the mutations mutR1 and mutR3 in GcvB. Nts of GcvB shaded in grey correspond to the conserved single-stranded regions R1 and R3. MicA sequence and its base-pairing interaction with *phoP* are in grey. (C) Compensatory changes in GcvB and *phoP-lacZ* RNAs restore control *in vivo*. The ß-galactosidase activity of strains deleted for *gcvB* and carrying either a wt *phoP-lacZ* fusion (strain MG1585) or its mutR3 derivative (strain MG1586) under control of the P_BAD_ promoter was assessed upon overexpression of MicA, GcvB or their derivatives. In the course of the experiment, RNA was extracted from the different transformants and the levels of GcvB, MicA and SsrA (used as a loading control) were analyzed by Northern-Blot. (D) A mutation in the R1 region of GcvB decreases its ability to regulate *livJ*. The ß-galactosidase activity of a P_BAD_-*livJ-lacZ* fusion (strain AC0067) was measured after transformation with GcvB or GcvBmutR1 overexpressing plasmids.

We had previously constructed a mutant form of *phoP-lacZ* (*phoPmut-lacZ*, where the 4 nts directly downstream of the AUG start codon are changed from CGCG to GCGC, Figure 3A) such that this fusion is no longer controlled by MicA [24]. Interestingly, this mutant fusion is still controlled by GcvB, since its expression is up-regulated by 2.2-fold in a *ΔgcvB* strain (Figure 2B, two last bars). This result suggests that the precise regions of the *phoP* mRNA that are required for MicA or GcvB action are different. Thus, GcvB acts on *phoP*, independently and apparently at a different site from that of MicA.

### The R1 Region of GcvB Is Not Required for the Control of *phoP*

GcvB is a pleiotropic regulator, whose expression is highest in exponentially growing cells in rich medium as a result of its control by the GcvA transcriptional regulator. GcvB directly regulates more than 20 genes, the large majority of which are targeted via a very well conserved single stranded G/U rich region of GcvB, referred to as R1 (Figure 3A). Even though this might be partially due to an experimental bias given that the R1 region was used as a “bait” in a bioinformatic search for targets, this region is clearly required for the control of almost all targets identified so far [13]. Since sRNAs often regulate multiple targets via a single conserved region [18], [29], [30], we reasoned that the R1 region was also likely to be involved in the control of *phoP*, regardless of whether *phoP* was a direct or indirect target. We therefore measured the expression of the P_BAD_-*phoP-lacZ* fusion in the presence of a plasmid overexpressing either GcvB wt or a GcvB mutant in the middle of the R1 region (GcvBmutR1, see Figure 3B). In this experiment, the chromosomal copy of *gcvB* is deleted and the steady-state level of GcvBmutR1 was slightly lower than that of GcvB wt. Somewhat surprisingly, these two forms of GcvB repressed the expression of *phoP-lacZ* to a similar extent (Figure 3C, left panel); in contrast, a previously identified target of GcvB R1 region, *livJ*, was, as expected, less regulated by GcvBmutR1 than by GcvB wt (Figure 3D). While this does not completely rule out a possible role for the R1 region in the control of *phoP* (for instance, if pairing involves nts of R1 that are not affected by the mutR1 change or if alternative pairing(s) can take place with this mutant), this suggests that the role of R1 in *phoP* control is not as crucial as for the other targets of GcvB.

### GcvB Is a Direct Regulator of *phoPQ*

Because GcvB action on *phoP* was independent of MicA (see above), we next envisioned the possibility that it could directly pair with *phoP* to control its expression. If this interaction exists, we expected it to involve a region of GcvB outside of the R1. The TargetRNA program [31] was used to predict a potential pairing between GcvB and the *phoP* mRNA fragment encompassing nts −36 to +30 relative to the AUG (i.e. the region of *phoP* that is present on the *phoP-lacZ* fusion). According to this prediction (Figure 3B), the region between nts 148 and 174 of GcvB can imperfectly pair with *phoPQ* mRNA in the translation initiation region (TIR), which is the most frequent binding site for negatively acting sRNAs. Interestingly, this corresponds to a region of GcvB that was shown to be mostly single-stranded in solution [12] and is now referred to as region R3. The relevance of this putative direct interaction was tested *in vivo* by compensatory changes. While the P_BAD_-*phoP-lacZ* fusion was repressed by more than 4-fold upon overproduction of GcvB wt, this was not the case with the GcvBmutR3 variant, where nts 154 to 158 were changed from CUGUC to GACAG. Rather, expression of the fusion was increased by more than 2-fold (Figure 3C). The inability of GcvBmutR3 to repress *phoP-lacZ* expression is not due to an intrinsic instability, since it accumulates to a level similar to that of wt GcvB (Figure 3C). A possible explanation for the fact that GcvBmutR3 activates *phoP-lacZ* is that it could titrate Hfq when overexpressed, leading to changes in expression of Hfq-regulated genes, such as *phoP* (see [28], [32] for examples of competition for Hfq). In contrast, GcvBmutR3, but not wt GcvB, caused an 8-fold decrease in the activity of the compensatory mutant fusion (Figure 3C, right panel), clearly showing that GcvB and *phoP* mRNA directly interact *in vivo*. It is also worth noting that MicA efficiently repressed both the wt and the mutant fusion, which confirms that GcvB and MicA pair at different loci of *phoPQ* mRNA. In addition, when mutations in the R1 and R3 regions of GcvB were combined, the resulting GcvBmutR1R3 repressed the expression of *phoPmutR3-lacZ*, but not that of *phoP-lacZ* (Figure S1A). This again indicates that, at least when GcvB is overexpressed, its R1 region is not involved in the control of *phoP*.

### *phoP* mRNA Interacts *In Vitro* with MicA and GcvB

**Figure 4:**
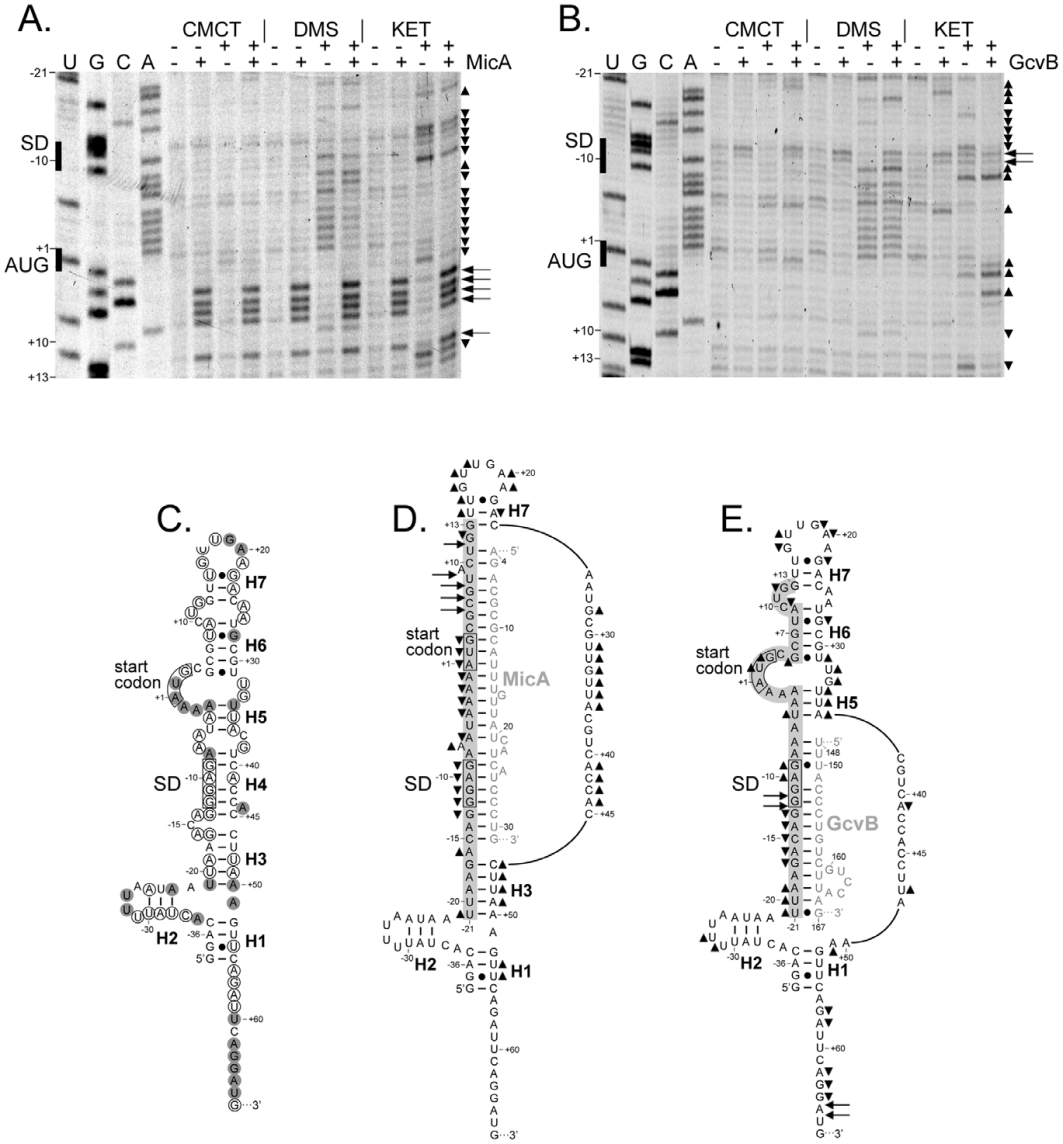
*In vitro* structural probing of *phoP* mRNA alone and in the presence of MicA or GcvB. (A, B) Representative gels showing structure probing of *phoP* mRNA using DMS, CMCT and kethoxal (KET) in the presence and absence of MicA (A) or GcvB (B) sRNA. Nucleotides are numbered according to position +1 which corresponds to the first nucleotide of *phoP* ORF. The locations of the SD sequence and *phoP* translation start codon are shown on the left of the gels. Up and down triangles on the right of the gels indicate increased or decreased reactivity upon sRNA addition, respectively, while the arrows denote sRNA-induced RT-stops or pauses. (C) Secondary structure model of the 5′ region of *phoP* mRNA. Nucleotides are numbered as in (A, B). Position −36 indicates the transcription initiation site from the P_1_ promoter. The seven double-stranded elements which hold the secondary structure of the 5′ region of *phoP* mRNA are denoted by H1 to H7. Nucleotides displaying weak and moderate or strong reactivity towards the chemical probes are indicated by empty or grey circles, respectively. The SD sequence and *phoP* translation start codon are boxed. (D, E) Summary of the reactivity shifts displayed by *phoP* mRNA nucleotides upon addition of MicA (D) or GcvB (E). The duplex models are derived from the base-pairing interactions shown in Figure 3 which have been altered according to the structural constraints provided by the probing data. The SD sequence and *phoP* translation start codon are boxed. The *phoP* mRNA nts highlighted in grey correspond to the region −21 to +13 whose probing is shown in panels A and B. sRNA-induced reactivity shifts and RT-stops or pauses are indicated as above (A, B). Nucleotides of both sRNAs are indicated in grey.

To provide experimental support to the proposed *phoP*-MicA and *phoP*-GcvB base-pairing interactions (Figure 3B), a structural probing analysis of *phoP* mRNA alone or in the presence of either sRNA was performed *in vitro* using chemical probes (Figure 4, A and B). DMS (dimethyl sulfate), CMCT (1-cyclohexyl-3-(2-morpholinoethyl)carbodiimide metho-p-toluene sulfonate) and kethoxal (1-1-dihydroxy-3-ethoxy-2-butanone) respectively modify unpaired adenosine (and to a much lesser extent cytidine), uridine and guanosine residues. According to our probing data, the secondary structure of the 5′ region of *phoP* mRNA appears as a long irregular stem-loop which is hold by seven double-stranded elements named H1 to H7, separated by bulges or loops (Figure 4C). Upon addition of MicA, most nts from positions −13 to +11 of *phoP* mRNA, which include the nts forming the 5′ strands of H4, H5 and H6, display either a decreased reactivity towards the probes or correspond to RT-stops or -pauses which occur in a region rich in GC pairs (Figure 4, A and D). This model is also consistent with the fact that many nts located between positions +27 to +45 of *phoP* mRNA, which include all the nts forming the 3′ strands of H4, H5 and H6 in the absence of MicA, become more reactive in the presence of MicA (Figure 4, A and D). In conclusion, the interaction between *phoP* mRNA and MicA relies on (i) the disruption of at least three of these elements, namely H4, H5 and H6, and (ii) the formation of an extended base-pairing interaction between nts −15 to +11 of *phoP* mRNA and nts 4 to 31 of MicA, whereby both the Shine-Dalgarno (SD) sequence and the *phoP* translation start codon are base-paired (Figure 4D). Upon addition of MicA, further reactivity enhancements in *phoP* mRNA nts are observed outside of the proposed *phoP* mRNA-MicA duplex (see nts +53 and +54 in H1, −21 and +46 to +49 in H3, −16 which joins H3 to the duplex, +14 to +21 in H7 and its apical loop, Figure 4D). It is likely that these changes are due to either local breathing or even disruption of H1, H3 and H7, which are destabilized by the binding of MicA. Also, decreased reactivities are observed (see nts +12 which joins the duplex to H7 and +23 in H7, Figure 4D) for which we have no explanation.

In contrast, the duplex formed by *phoP* mRNA and GcvB seems shorter as it requires only the disruption of H3 and H4 to form; it is centered around and blocks the SD sequence, which is in complete agreement with the *in vivo* data. Indeed, nts displaying decreased reactivities towards the probes or corresponding to a region where GcvB-induced RT-stops or -pauses occur are clustered between nts −17 and −11 of *phoP* mRNA (Figure 4B and 4E). However, the duplex is likely to be subject to breathing as a certain number of nts located on both side of the cluster become more reactive in the presence of GcvB (see nts −21 to −19, −10 and −9, Figure 4E). Additional reactivity enhancements have been mapped outside of the proposed duplex (see nts −5, +2, +3, +5 and +31 to +36 in H5 and H6 and in the bubble located in between, Figure 4E), which are probably due to breathing of H5 which results from its destabilization by the binding of GcvB. Other regions of *phoP* mRNA located outside of the predicted duplex are subject to increase or decrease in reactivity in the presence of GcvB (see nts located in H6 and H7 and in the bubble in between, Figure 4E). They can be due to some rearrangement of the overall structure of *phoP* mRNA upon GcvB-binding and/or to a supplementary interaction between *phoP* mRNA and GcvB. For instance, nts +9 to +17 of *phoP* mRNA, several of which appear protected upon GcvB-binding, could theoretically pair with nts 89 to 97 of GcvB. While our *in vivo* data show that this putative supplementary interaction is not sufficient for control, it could nevertheless play a role in stabilizing the *phoP* mRNA-GcvB duplex or in increasing the kinetics of association. At this stage, its existence and importance remains to be experimentally addressed. Finally, the reactivities of nts +37 to +50, which form the 3′ strands of H3 and H4 in the absence of GcvB, could not be assessed with confidence because of the presence of several RT-stops or –pauses which are also present when *phoP* mRNA alone is reverse-transcribed in the absence of the probe (data not shown).

### Both MicA and GcvB Decrease the Level of *phoPQ* mRNA

**Figure 5:**
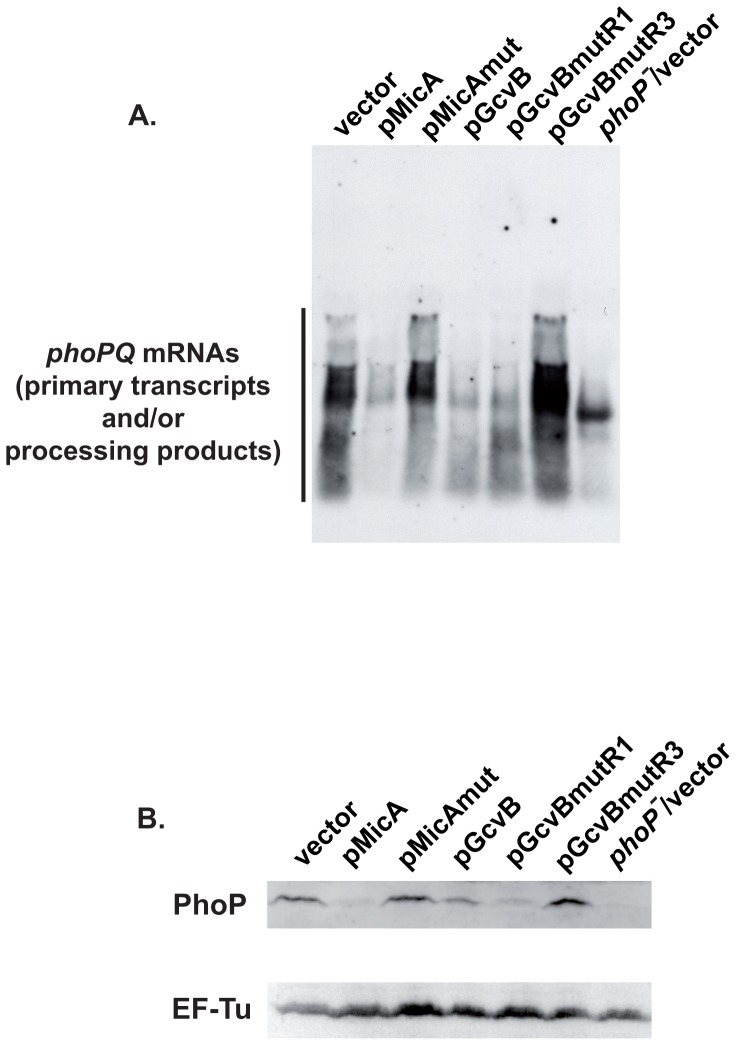
The control of *phoP* by regulatory sRNAs is accompanied by a strong decrease in the steady-state level of *phoPQ* mRNA, but not always of PhoP protein. (A) The level of *phoPQ* mRNA was monitored by Northern-Blot upon overproduction of MicA, GcvB and their derivatives. Strains used in this experiment are MG1516 and MG1517, where the wt *phoPQ* operon (MG1516) or the *phoPQ* operon interrupted by the insertion of a kanamycin resistance cassette within *phoP* (MG1517) were put respectively under control of the P_BAD_ promoter. (B) Western-Blot analysis of PhoP protein upon overproduction of MicA, GcvB and their derivatives. Strains are MG1173 (*phoP^+^*) or MG1446 (*phoP^−^*). The level of EF-Tu was monitored and used as a loading control.

Target-mRNAs of negatively acting Hfq-dependent sRNAs are frequently degraded upon sRNA production. Therefore, to confirm the results obtained above by gene fusion, the levels of *phoPQ* mRNA were analyzed by Northern-Blot upon overexpression of MicA, GcvB or their mutant derivatives, using a chromosomal P_BAD_-*phoPQ* construct (Figure 5A). In this experiment, transcription of the *phoPQ* operon is again expected to start 36 nts upstream of the *phoP* start codon and has been put under the control of the P_BAD_ promoter for two reasons: (i) to focus only on promoter-independent regulation and (ii) because of the low abundance of the *phoPQ* mRNA when expressed from its own promoters under the experimental conditions used here. With this construct, several specific bands are visible. The upper band migrates below a 3 kb RNA marker and most likely corresponds to the whole *phoPQ* mRNA, while the bands of lower molecular weight could result from either alternative transcription or processing events (Figure 5A). MicA, GcvB and GcvBmutR1 induce a decrease in *phoPQ* mRNA levels, but not MicAmut and GcvBmutR3, that have lost the ability to control *phoP* expression. This is in complete agreement with the results obtained with the *phoP-lacZ* fusions. Pairing of MicA and GcvB to the *phoPQ* mRNA is therefore likely to induce a degradation of this mRNA.

### MicA and GcvB Differentially Affect the Level of PhoP Protein

The effect of MicA or GcvB on the steady-state levels of the PhoP protein was then investigated by Western-Blot analysis, in a strain where *phoPQ* is expressed from its own promoter. As expected, MicA overexpression resulted in a strong decrease in the amount of PhoP (Figure 5B), while overexpression of MicAmut had no noticeable effect. When GcvB was overexpressed, PhoP levels were decreased, albeit to a much lesser extent than upon MicA overproduction. This is rather surprising since pMicA and pGcvB had a similar effect on the expression of *phoP-lacZ* (Figure 1, Figure 2, and Figure 3) and on the levels of *phoPQ* mRNA (Figure 5A). Interestingly, pGcvBmutR1, whose effect was also similar to that of pMicA and pGcvB in the previous experiments, is more efficient than pGcvB in down-regulating the levels of PhoP protein. Finally, GcvBmutR3 overproduction had no effect on the levels of PhoP, which is consistent with its inability to repress *phoP* expression (Figure 5B).

Therefore, control of *phoP* by GcvBmutR1 or MicA results, as expected, in a clear decrease of the PhoP protein levels. Surprisingly however, this decrease is only modest with wt GcvB, most likely because its R1 region has pleiotropic effects in the cell under the conditions used here, as discussed below.

### MicA and GcvB Differentially Affect Expression of the PhoP Regulon

**Figure 6:**
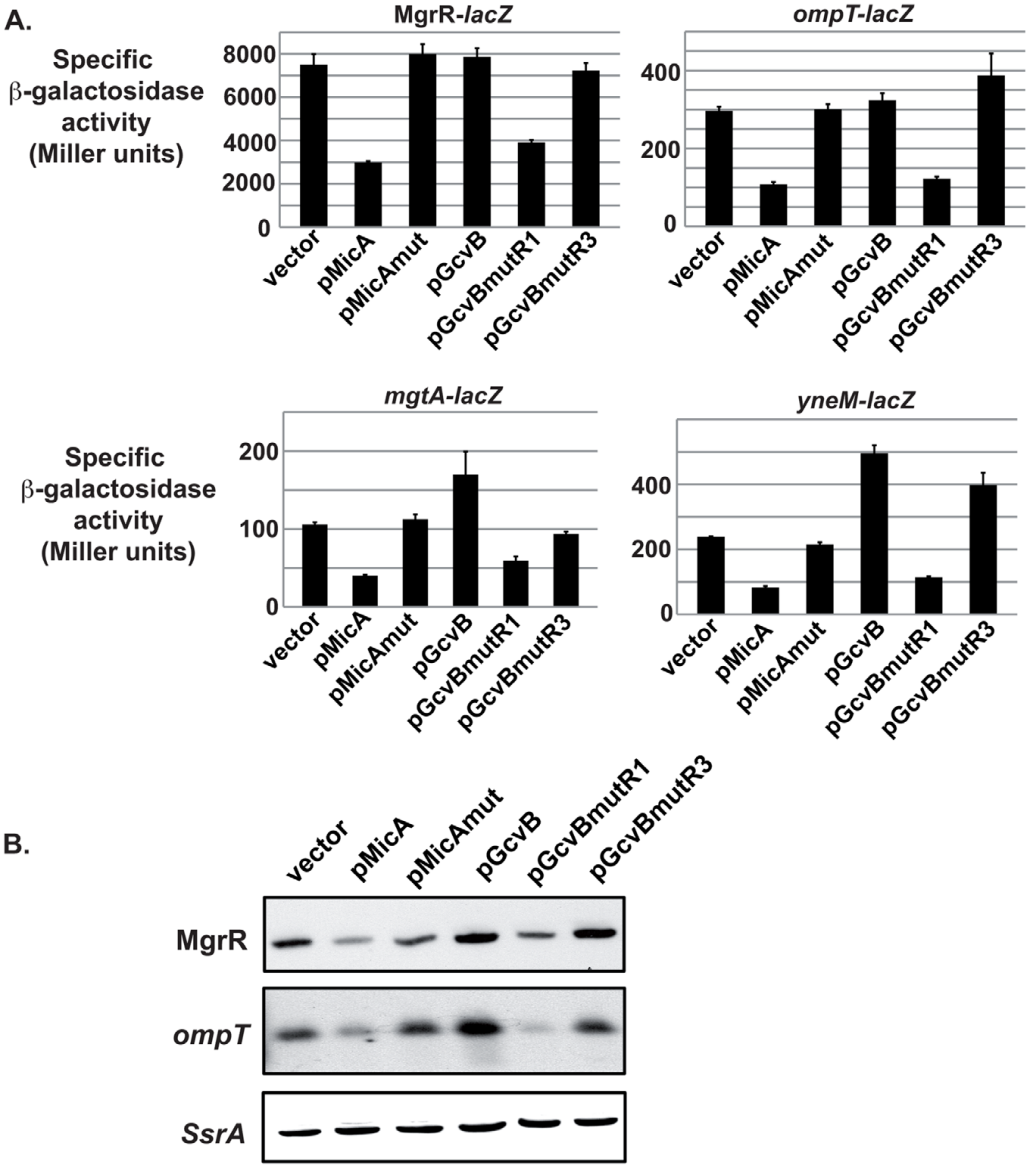
MicA and GcvB differentially affect expression of the PhoP regulon. The effect of overproducing MicA, GcvB and their mutants on the expression of several PhoP-regulated genes was analyzed by gene fusion with *lacZ* (A) or by Northern-Blot (B). Strains used in panel A are MG1173, MG1528, KM112 and KM194, which carry respectively *ompT*-, *mgtA*-, *mgrR*- and *yneM*-*lacZ* fusions. For panel B, strain MG1425 was transformed by plasmids overexpressing different sRNAs, grown to mid-log phase and RNA was extracted to monitor the levels of *ompT* and MgrR by Northern-Blot. SsrA detection was used as a loading control.

We then tested whether MicA and GcvB can control the expression of the PhoP regulon by repressing PhoQ-PhoP synthesis. For this purpose, the expression of 4 genes whose transcription is directly activated by PhoP [25] was analyzed under conditions where either MicA or GcvB was overproduced (Figure 6). These 4 genes are *ompT*, *mgtA*, *yneM* and *mgrR*, that encode an outer membrane protease, a magnesium transporter, a protein of unknown function located in the outer membrane and a sRNA regulator of LPS modification respectively. As expected, MicA induced a >2.5-fold decrease in expression of these 4 targets as analyzed by either translational fusions (for *ompT*, *mgtA*) or by transcriptional fusions (for *mgrR* and *yneM*) to (Figure 6A). This decrease is most likely due to *phoP* regulation, since MicAmut, that does not regulate *phoP*, does not affect expression of these target genes. Similarly, the overproduction of GcvBmutR1, that also represses *phoP*, led to a ∼2-fold decrease in the expression of the 4 fusions, while overexpression of GcvBmutR3 does not, in agreement with its inability to control *phoP*. Finally, when the same experiment was carried out in the GcvB overproducing strain, no decrease was observed in the expression of the 4 members of the PhoP regulon that were tested. Instead, activity of *mgrR*- and *ompT-lacZ* was unchanged, while activity of *mgtA*- and *yneM-lacZ* was increased by 1.6 and 2.1-fold respectively (Figure 6A). These results obtained by gene fusion were confirmed when the levels of *ompT* or MgrR RNAs were analyzed by Northern-Blot (Figure 6B). Indeed, MicA and GcvBmutR1 induced a decrease in the level of both RNAs, whereas MicAmut, GcvB wt and GcvBmutR3 did not.

Therefore, MicA and GcvBmutR1 repress the PhoP regulon, by controlling expression of *phoP*. However, wt GcvB does not, which is consistent with the only modest decrease observed in PhoP levels upon its overproduction.

### MicA and GcvB Inhibit Ribosome Binding to the *phoP* TIR

**Figure 7:**
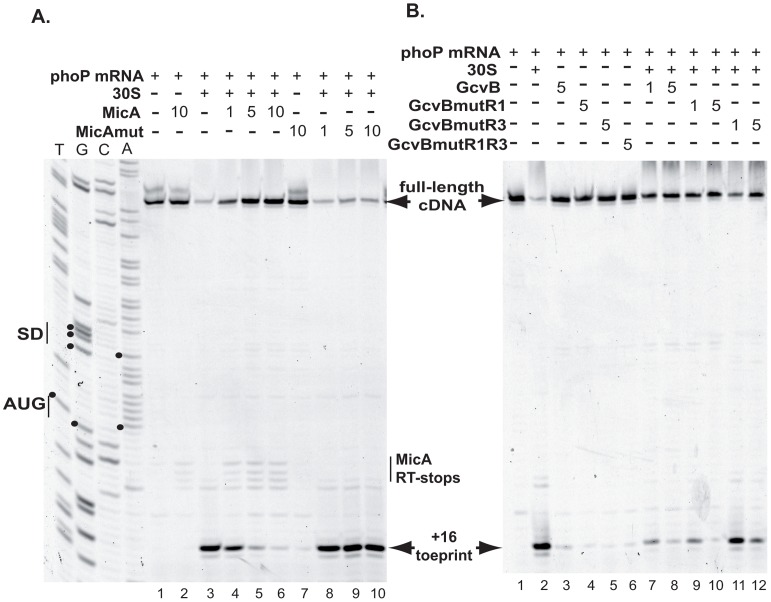
MicA and GcvB repress *phoP* translation through inhibition of ribosome binding. Toeprinting experiments were performed in presence of MicA and MicAmut (panel A) or GcvB, GcvBmutR1 and GcvBmutR3 (panel B) sRNAs. The relative amount between sRNAs and *phoP* mRNA are given above the gel. The full-length cDNA as well as the arrest of reverse-transcription induced by ribosome binding (+16 toeprint) are indicated by arrows. The pauses in RT induced by MicA binding to *phoP* mRNA are indicated by “MicA RT-stops”. Positions of the SD sequence and AUG start codon of *phoP* are shown on the sequencing lanes.

In most cases, negatively acting sRNAs base-pair with their target-mRNAs in the TIR and occlude the RBS, thereby preventing ribosome binding and translation initiation. This is frequently accompanied by a degradation of the target-mRNA, possibly as a consequence of translational block, or in a process that is directly induced by the sRNA pairing to the target-mRNA. Since MicA and GcvB both pair to *phoPQ* mRNA in the TIR of the first cistron and decrease *phoPQ* mRNA levels, toeprinting experiments were performed in order to determine whether they also inhibited ribosome binding. In these experiments, addition of 30S ribosomal subunit and initiator tRNA to a ∼200-nt *phoP* mRNA fragment transcribed *in vitro* induced an arrest of reverse-transcription, that was visible on a sequencing gel as a band at position +16, a classical toeprint position (Figure 7, A and B, lanes 3 and 2 respectively). When increasing concentrations of MicA were incubated with *phoP* mRNA prior to the addition of 30S and fMet-tRNA, the intensity of this band progressively decreased (Figure 7A, lanes 4–6). This was not observed when equal amounts of MicAmut were added instead (lanes 8–10), suggesting that it is the pairing of MicA to *phoP* TIR that inhibits ribosome binding. Similar results were observed with GcvB, whose addition inhibited the appearance of the +16 toeprint, even more efficiently than MicA (Figure 7B, lanes 7–8). Again, this is most likely due to the pairing between GcvB and *phoP* since GcvBmutR1, that should still pair with *phoP*, also inhibited the toeprint, while GcvBmutR3 and GcvBmutR1R3, that should not bind to *phoP*, inhibited the toeprint much less efficiently (Figure 7B, lanes 11–12, and Figure S1B). It is interesting that GcvB is much more efficient than MicA in inhibiting toeprint, and that GcvBmutR3 still inhibits toeprint to some extent. This might be related to the existence of a bipartite interaction between *phoP* and GcvB (see above).

Furthermore, as already observed in probing experiments (Figure 4), addition of MicA, but not MicAmut, to *phoP* mRNA in the absence of 30S subunits also induced stops or pauses of reverse-transcription, as indicated by the bands at positions +6 to +8 (Figure 7A, lanes 2, 4, 5 and 6). This corresponds to the 3′ end of the duplex between MicA and *phoP* (Figures 3A, 4D, [24]), indicating that this duplex is stable enough to induce pauses in reverse-transcription. In contrast, while GcvB pairs with *phoP in vitro*, given the results of the probing experiments and the toeprint inhibition, its binding does not induce pauses or stops of the reverse transcription that are sufficiently strong to be observed in this experiment. This is in contrast to what was observed in Figure 4 and this discrepancy is most likely due to the use of different experimental conditions in the probing and toeprint experiments. In fact, under conditions where the signal is highly amplified, reverse transcriptase stops are visible in the toeprint experiments (data not shown). Hfq protein was not included in these *in vitro* assays, because of the risk of non-specific interactions with RNA. The fact that, even in the absence of this chaperone, MicA and GcvB could both pair to *phoP* mRNA *in vitro* (i.e. in the absence of RNases) suggests that the requirement for Hfq *in vivo* is, at least in part, explained by its ability to protect MicA and GcvB from degradation.

In summary, both MicA and GcvB inhibit ribosome binding by pairing to *phoPQ* TIR. This translational block could be the step leading to the degradation of the target-mRNA in presence of the regulatory sRNAs observed *in vivo*.

## Discussion

### GcvB as a Novel Regulator of *phoP* Expression: A Control Likely Conserved in Several Enterobacteria

In this study, we identify *phoPQ* mRNA as a new target of the *E. coli* GcvB sRNA. After MicA, this is thus the second sRNA regulator of this operon. Similar to MicA, GcvB directly controls *phoPQ* expression by pairing to the TIR of *phoP*, although at sequences slightly different from those of MicA. These pairings cause a steric inhibition of ribosome binding as seen by toeprint experiments and, possibly as a consequence of this translational control, induce degradation of the *phoPQ* mRNA. Furthermore, this work identifies a novel pairing region of GcvB, namely R3, essential for *phoP* control. Interestingly, this region was predicted in a computational approach as a potential target-binding region of GcvB (together with the R1) on the basis of its conservation and accessibility [33], and was proposed to participate in the control of *cycA* expression [34]. Whether GcvB controls yet additional genes through its R3 region remains to be investigated.

This region R3 is with R1 and R2 one of the most conserved in GcvB among enterobacteria (Figure S2A). However, *phoP* was not identified as a GcvB target in *Salmonella* in a recent study combining microarray analysis following GcvB pulse-expression and bioinformatic prediction based on complementarity to the R1 region [13]. While this could be due to the low abundance of *phoPQ* mRNA and to the fact that this control does not rely on R1, this could also indicate that the control of *phoP* by GcvB that exists in *E. coli* is not conserved in *S. typhimurium*. Consistent with this, predictions of pairing between GcvB R3 region and the TIR of *phoP* mRNA in *Salmonella* identified only 4 consecutive complementary nts at the most, which is probably too short to ensure specific binding. Furthermore, a preliminary analysis of potential interactions between GcvB R3 and the *phoP* TIR in different families of enterobacteriaceae suggests that GcvB could control *phoP* in species such as *Klebsiella pneumoniae*, *Photorhabdus luminescens*, *Proteus mirabilis*, *Serratia proteamaculans*, *Shigella flexneri*, *Xenorhabdus bovienii* and *Rahnella* (Figure S2B).

### Why Does wt GcvB, but Not GcvBmutR1, Only Modestly Affect PhoP Protein Levels?

At first glance, it is quite surprising that, even though MicA, GcvB and GcvBmutR1 similarly repress *phoP* expression when followed by gene fusion to *lacZ* or mRNA levels, their effects on the PhoP protein are quite different. Indeed, while MicA and GcvBmutR1 strongly decreased the cellular level of PhoP, as expected, the effect of wt GcvB was much more moderate (Figure 5B). One noticeable difference in those experiments is that when the PhoP protein levels were assessed, *phoPQ* was expressed from its own promoter. In contrast, in both the experiments with gene fusion or mRNA levels, its transcription is driven by an heterologous promoter (P_BAD_), with *phoPQ* and *phoP-lacZ* mRNAs expected to originate at the same transcription start site than from P_1_. Therefore, one could hypothesize that wt GcvB would activate PhoP synthesis from transcripts originating from promoters P_2_ or P_3_ (upstream of P_1_), or by acting on *phoPQ* transcription, in addition to repress its expression post-transcriptionally. These possibilities were experimentally ruled out because (i) wt GcvB similarly repressed expression of a *phoP-lacZ* fusion whose 5′end is identical to the 5′end initiating from P_1_ or from P_2_ promoter (Figure S3A) and (ii) wt GcvB only poorly affects *ompT* expression (and even increases *yneM* expression) in a strain where all the *phoPQ* promoters have been replaced by P_BAD_ (Figure S3B).

One could also envision that, when expressed from its own promoter under non-inducing conditions (as in Figure 5B), *phoP* expression is poorly affected by wt GcvB, whose R1 region can pair with other competing targets. This competition could not take place with GcvBmutR1, where the R1 region is mutated, in agreement with its ability to control *phoP* in all experiments. In contrast, when *phoP* expression increases, for instance because its transcription is driven by an induced P_BAD_ promoter, it would now become available for repression by wt GcvB, because it would outcompete other GcvB targets, hence the stronger effect of wt GcvB on *phoP* in Figures 3C and 5A for instance. It will now be interesting to study how the induction of *phoP* from its own promoter and the expression of GcvB R1 targets will impact the control of *phoP* by GcvB; in other words, whether this model is physiologically relevant.

Yet another possibility to explain the difference between *phoP-lacZ* expression, *phoPQ* mRNA and the PhoP protein levels in presence of pGcvB is that, in addition to its negative effect on *phoP* expression at the translation initiation step, wt GcvB could stabilize the PhoP protein. Such a regulatory event would affect only PhoP levels, but not the activity of *phoP-lacZ* or *phoP* mRNA levels. This putative stabilization would be dependent on the R1 region of GcvB and could be mediated by one or several of its targets. Furthermore, one could wonder whether this stabilization is related to the phosphorylation status of PhoP protein. Because these R1 targets that could directly or indirectly control PhoP are likely to be multiple, it might be difficult to identify them by a genetic approach. Again, it is tempting to speculate that the expression of these targets, as well as the availability of the R1 region to regulate them (dependent on the expression of all GcvB targets and their relative affinity for the sRNA), will play an important role in the control of the PhoP regulon by GcvB. While under our experimental conditions, the amount of PhoP protein is not strongly affected by wt GcvB, there might be conditions where it could be. This would provide a mechanism to establish a hierarchy among the different GcvB-targets and to monitor the regulatory outcomes of GcvB-mediated controls.

### A Novel Example of the Complex Interplay between sRNAs and Transcriptional Regulation Networks

**Figure 8:**
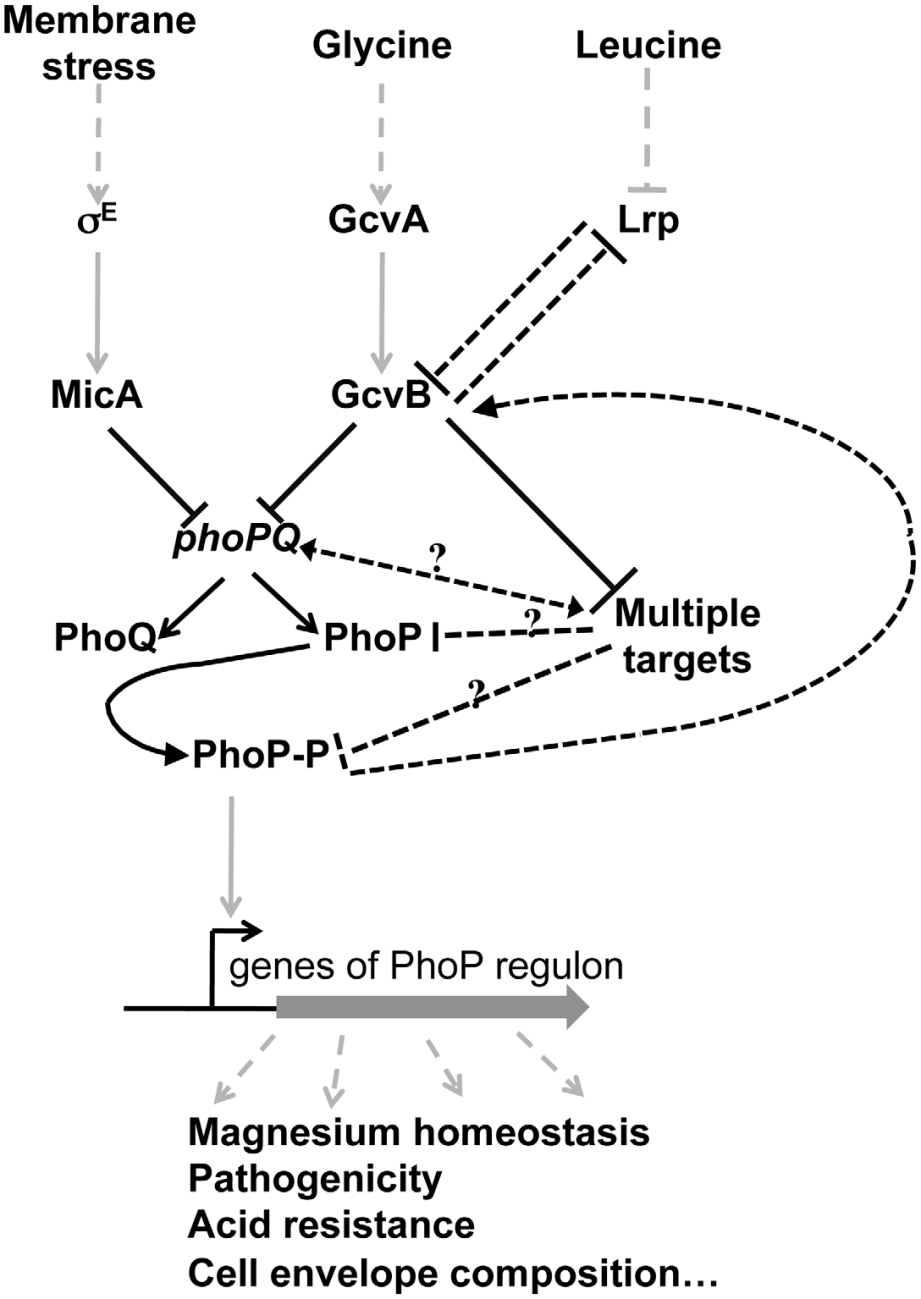
Model of regulation of *phoP* and PhoP regulon by MicA and GcvB sRNAs. Grey and black continuous lines indicate respectively transcriptional and post-transcriptional regulations. Dashed black lines refer to controls whose mechanism is still unclear (possibly indirect). Dashed grey lines indicate input signals and output effects. See text for details.

In a previous work, we showed that MicA, which is induced by envelope stress, repressed the expression of *phoPQ*. This finding related the activity of this operon to the cell envelope status [24]. Our present findings show that GcvB relates amino acid (or peptide) uptake and metabolism to the expression of *phoPQ*. The induction of GcvB occurs in the presence of two different amino acids with two different mechanisms. First through the GcvA/GcvR repressing complex that is inactivated in the presence of glycine, and second through the global regulator Lrp, whose repression is alleviated in the presence of leucine [9], [11] (Figure 8). Although the *raison d'être* of such a connection between amino acid uptake/metabolism and PhoQ/PhoP regulon activity is not obvious, such a relationship has already been observed with several targets of this regulon. For instance, expression of *mgtA* and *mgtCBR* genes is derepressed under conditions of proline limitation due to the presence of a proline-rich open reading frame in their leader mRNAs [35], [36]. Another example is the proline transporter encoded by the *proP* gene that is also regulated by PhoQ/PhoP [37].

The complexity of the relationship between GcvB and the *phoPQ* regulon is highlighted by two experimental data. The first is the surprising way wt GcvB fails to strongly decrease PhoP levels as discussed above. The second is related to the recent results of a deep-sequencing study indicating that PhoP positively regulates GcvB levels in the cell [38](Figure 8). However, GcvB levels were unmodified when the magnesium concentration in the growth medium was varied. Therefore, GcvB could also modulate the degree of PhoQ/PhoP activation depending on the inducing signals.

Interestingly, there are already many examples of connections between sRNAs and TCS, as several sRNAs were previously shown to control TCS and conversely [39]. In addition, the negative feedback loop that exists between *phoP* and GcvB is reminiscent of other feedback loops involving sRNAs [40]. As in most cases however, the properties and possible advantages of this feedback loop in bacterial physiology remain to be experimentally addressed.

Our search for Hfq-dependent sRNAs regulators of the PhoQ/PhoP TCS was initially motivated by the fact that *phoP* expression was up-regulated in an *hfq* mutant strain independently of MicA. Interestingly however, even though GcvB is partially responsible for this Hfq-effect, expression of *phoP* is still higher in an *hfq* mutant than in *hfq^+^* cells in the absence of both MicA and GcvB (data not shown). This suggests that there might be even more sRNAs controlling PhoQ/PhoP, which would allow the integration of yet additional signals to fine-tune expression of this central TCS.

## Materials and Methods

### General Microbiological Techniques and Strain Construction

Strains and plasmids used in this study are listed in Table 1, and sequences of the oligonucleotides in Table S1. Strains were grown aerobically in LB medium at 37°C. When needed, antibiotics were used at the following concentrations: ampicillin 150 µg/ml, tetracyclin 10 µg/ml, kanamycin 25 µg/ml or chloramphenicol 10 µg/ml. PCR amplification was performed using the Phusion DNA polymerase (New England Biolabs). IPTG (isopropyl-ß-D-thiogalactopyranoside) was used at a final concentration of 100 µM.

**Table 1:** Strains and plasmids used in this study.

Strains		
Name	Characteristics	Source
MG1655	Wild-type strain	F. Blattner
DJ480	MG1655 *Δlac*X174	D. Jin, NCI
DJ624	DJ480 *mal::lacI^q^*	D. Jin, NCI
NM300	DJ480 *mini-λ tet^R^*	N. Majdalani, NCI
NM1200	MG1655 *mini-λ cm^R^*	N. Majdalani, NCI
PM1205	MG1655 *mal::lacI^q^ ΔaraBAD araC^+^ lacI':: P_BAD_-cat-sacB-lacZ*, *mini-λ tet^R^*	[19]
MG1173	DJ624 λRS*ompT-lacZ*	[24]
MG1425	MG1655 *mal::lacI^q^ ΔaraBAD araC^+^* P_BAD_-*phoP-lacZ*	[24]
MG1428	MG1655 *mal::lacI^q^ ΔaraBAD araC^+^* P_BAD_-*phoP* ***mut*** *-lacZ*	This study
MG1430	MG1425 *ΔmicA::cm*	[24]
MG1446	MG1173 *phoP::kan*	[24]
MG1452	MG1425 *ΔmicA::tet*	[24]
MG1508	MG1655 *mal::lacI^q^ P_LtetO-1_-cat-sacB-lacZ*, *mini-λ tet^R^*	This study
MG1510	MG1655 *mal::lacI^q^ ΔaraBAD araC^+^* P_BAD_-*phoP* ***mutR3*** *-lacZ*	This study
MG1511	MG1655 *mal::lacI^q^ P_LtetO-1_-phoP-lacZ*	This study
MG1516	DJ624 Cm-P_BAD_-*phoPQ*	This study
MG1517	DJ624 Cm-P_BAD_-*phoP::kan*	This study
MG1521	MG1511 *ΔgcvB::kan*	This study
MG1528	MG1655 *mal::lacI^q^ P_1_-mgtA-lacZ*	This study
MG1585	MG1425 *ΔgcvB::kan*	This study
MG1586	MG1510 *ΔgcvB::kan*	This study
MG1709	MG1430 *ΔgcvB::tet*	This study
MG1768	MG1425 *ΔgcvB::tet*	This study
MG1769	MG1428 *ΔgcvB::tet*	This study
AC0067	MG1655 *mal::lacI^q^ ΔaraBAD araC^+^* P_BAD_-*livJ-lacZ*	This study
KM112	PM1205 *mgrR-lacZ* fusion	[43]
KM194	PM1205 *yneM-lacZ* fusion	[43]

Plasmids		
Name (short name)	Characteristics	Source
pBRplac (vector)	pBR322 derivative carrying a modified P_LlacO-1_ promoter, AmpR, TetR	[47]
pBRplacMicA (pMicA)	*micA* under modified P_LlacO-1_ in pBRplac, AmpR	[19]
placTetMicA (pMicA)	*micA* under modified P_LlacO-1_ in pBRplac, AmpR, TetR	This study
placTetMicAmut (pMicAmut)	*micAmut* under modified P_LlacO-1_ in pBRplac, AmpR, TetR	This study
pBRplacGcvB (pGcvB)	*gcvB* under modified P_LlacO-1_ in pBRplac, AmpR, TetR	[8]
pBRplacGcvBmutR1 (pGcvBmutR1)	*gcvBmutR1* under modified P_LlacO-1_ in pBRplac, AmpR, TetR	This study
pBRplacGcvBmutR3 (pGcvBmutR3)	*gcvBmutR3* under modified P_LlacO-1_ in pBRplac, AmpR, TetR	This study
pBRplacGcvBmutR1R3 (pGcvBmutR1R3)	*gcvBmutR1R3* under modified P_LlacO-1_ in pBRplac, AmpR, TetR	This study

Replacement of *gcvB* gene by a kanamycin or tetracyclin resistance cassette was engineered by recombineering of a cassette amplified by PCR (with ΔgcvB::kanfor and rev, or ΔgcvB::tetfor and rev oligonucleotides) and flanked by homology regions upstream and downstream of *gcvB* into a strain carrying a mini-lambda allowing recombineering upon induction, such as NM300 or NM1200 for instance. These mutant alleles, as well as *ΔmicA::tet* [24] or *phoP::kan* [41] were then moved by P1 transduction when necessary.

Strains carrying gene fusions to *lacZ* were either obtained from different sources or constructed in this study by recombineering into strain PM1205 [19] or strain MG1508. In strain MG1508, a *cat-sacB* cassette following the P_LtetO-1_ promoter [42] is placed upstream of the *lacZ* gene in an MG1655 derivative that carries a mini-lambda.

Strain MG1173 carries a translational *ompT-lacZ* fusion, whose construction was described previously, at the lambda attachment site [18]. Strains KM112 and KM194 were also described elsewhere [43]. They contain respectively the promoter region up to nt+10 of MgrR, or the promoter region of *yneM* up to nt+91 (nt+1 is the transcriptional start site) upstream of *lacZ* chromosomal gene, starting 17 nts upstream of its ATG start codon. The translational P_tet_-*phoP-lacZ* fusion was constructed by replacing the *cat-sacB* cassette of the P_tet_-*cat-sacB-lacZ* construct in strain MG1508 with a PCR fragment encompassing nts −36 to +30 (relative to the ATG start codon) of *phoP* between homology regions to P_tet_ and *lacZ* respectively. This PCR fragment was generated with primers 5′Ptet-phoP and 3′phoP-lacZ. Recombinants were selected on LB-agar plates without NaCl supplemented with 6% sucrose, and further verified as in [24]. Similarly, construction of the P_1_-*mgtA-lacZ* fusion was done by recombineering of a PCR fragment carrying nts −330 to +30 of *mgtA* in MG1508, except that the homology regions were upstream of Ptet and within *lacZ* (see primers 5′P_1_mgtA and 3′mgtA-lacZ).

For strains MG1510 (P_BAD_-*phoPmutR3-lacZ*) and AC0067 (P_BAD_-*livJ-lacZ*), PCR fragments were generated with primers phoPmutR3for and 3′phoP-lacZ, or 5′LivJ-lac and 3′LivJ-lac respectively, then recombined into strain PM1205.

Construction of strains where the *phoPQ* operon, wt or interrupted by a kanamycin resistance cassette, is expressed from a P_BAD_ promoter was as follows. First, the chloramphenicol resistance cassette followed by the P_BAD_ promoter was amplified by PCR from plasmid pTM26 [44] with primers 5′Cm-PBAD-phoPQ and 3′Cm-PBAD-phoPQ. This product was then recombined in strain NM300 (or a derivative carrying the *phoP::kan* allele moved by P1 transduction using AB043 [41] as the donor strain). After selection on LB-chloramphenicol plates, the P_BAD_ promoter and beginning of *phoP* gene were checked by sequencing, and resistance to kanamycin was verified for the P_BAD_-*phoP::kan* construct. These alleles were then moved by P1 transduction using DJ624 as the recipient strain and selecting for chloramphenicol resistant clones to create strains MG1516 and MG1517. MG1517 was checked for resistance to kanamycin.

### Plasmids

#### MicA overexpressing plasmids

The pBRplacMicA plasmid was previously described (pMicA in [24]): MicA was cloned between AatII and HindIII restriction sites so that the resulting MicA overexpressing plasmid does no longer confer tetracyclin resistance. In the present study, it was preferable to use tetracyclin resistant plasmids for some of the experiments because of the growth defects observed with pBRplacGcvB. Therefore, the placTetMicA or its MicAmut derivative were constructed that carry *micA(mut)* under the modified P_LlacO-1_ promoter and that are both ampicillin and tetracyclin resistant. Briefly, micA(mut) gene was PCR amplified from pBRplacMicA(mut) plasmid with primers pBRfor and 5′Hind-MicA-tet. The PCR products were then digested with AatII and HindIII and ligated in pBRplac cut with the same enzymes. The resulting plasmids were checked by sequencing. More precisely, pMicA is pBRplacMicA in Figures 1, 2 and 5B; and it is placTetMicA in Figure 3, Figure 4, Figure 5A, and Figure S1.

#### GcvB overexpressing plasmids

The plasmid overexpressing wt GcvB is from P. Mandin [8]. Its mutR1 and mutR3 derivatives were constructed using the Quick-change site-directed mutagenesis kit (Stratagene) with primers GcvBmutR1for and GcvBmutR1rev, or GcvBmutR3for and GcvBmutR3rev respectively, following manufacturer's instructions. For the mutR1R3 mutant, the same kit was used with primers GcvBmutR3for and GcvBmutR3rev, but the template plasmid was pBRplacGcvBmutR1. After sequencing, the AatII-EcoRI fragment was cloned in pBRplac cut with the same enzymes to prevent any secondary mutations.

### ß-Galactosidase Assay

Overnight cultures were diluted 500 fold in fresh medium (see below for exact medium composition) and grown to mid-exponential phase (OD at 600 nm∼0.4). The ß-galactosidase activity was then measured as in [45] and expressed in Miller units. Alternatively, the activity was measured in a 96-wells plate for some experiments. In this case, 100 µl of cells were mixed with 50 µl of permeabilization buffer containing 200 µg/ml of polymixin B [46]. After addition of 50 µl of ONPG, the absorbance at 420 nm was followed over-time and the ß-galactosidase activity was calculated as the slope of the resulting curve. It is expressed in arbitrary units.

Cells were grown in the following media: for Figure 1A, LB-Ampicillin-IPTG-Arabinose 0.002% or 0.02%; for Figure 1B, LB-Ampicillin-IPTG; for Figure 1C, LB; for Figure 2A and 3C, LB-Ampicillin-IPTG-Arabinose 0.02%; for Figure 2B, LB-Arabinose 0.002%; for Figure 3B, LB-Ampicillin-IPTG-Arabinose 0.02%, but IPTG was not included in the overnight cultures; for Figure 6A, LB-Tetracyclin-IPTG; for Figure S1, LB-Tetracyclin-IPTG-Arabinose 0.02%.

Values of ß-galactosidase activity given in the paper are the average of at least two independent experiments and are listed in Table S2.

### RNA Extraction and Northern Blot Analysis

RNA was extracted following the hot phenol method as previously described [47] and using 650 µl of cells. For experiment of Figure 3, RNA was extracted at the same time than samples were taken to measure ß-galactosidase activity. For experiments of Figures 5 and 6, cells were grown overnight in LB-Tetracyclin-IPTG-Arabinose (0.2% for Figure 5 and 0.02% for Figure 6), then diluted in fresh medium and grown to mid-exponential phase (A_600_∼0.4 for Figure 6B) or to stationary phase (A_600_∼2 for Figure 5) before RNA was extracted. For Northern analysis, a constant amount of RNA was separated on an 8% acrylamide TBE-urea gel (for MicA, GcvB, MgrR and SsrA RNAs) or on a 1% denaturing agarose gel (for *ompT* and *phoP* mRNAs) and transferred to an Hybond-N+ membrane. Detection was then performed using biotinylated probes and the Ambion brightstar detection kit following manufacturer's instructions.

### Western Blot Analysis

Overnight cultures in LB-Tetracyclin-IPTG of strain MG1173 (wt) or MG1446 (*phoP^−^*), transformed with pBRplac and derivatives, were diluted 500-fold in the same medium and grown to mid-exponential phase. Cells were then pelleted and resuspended in SDS-sample buffer with DTT (New England Biolabs) at a final concentration of 15 OD_600_/ml. These samples were then boiled for 5 minutes and 15 µl were loaded on a 15% SDS-PAGE gel. Proteins were then transferred to an Hybond-C super membrane (Amersham) and the PhoP protein was detected using a 1∶1000 dilution of an anti-PhoP antiserum (from Mark Goulian) and the Immun-Star WesternC Chemiluminescent Kit (Biorad). EF-Tu was immunodetected from the same membrane. A representative blot from three independent experiments is shown.

### *In Vitro* RNA Transcription

PCR templates for the *in vitro* transcription of MicA or GcvB (and their derivatives) were prepared from the pBRplacMicA(mut) or pBRplacGcvB(mutR1, mutR3 or mutR1R3) plasmids, using the oligonucleotides 5′T7MicA(mut) and 3′T7MicA, or 5′T7GcvB and 3′T7GcvB respectively. Note that one or two G residues are added at the 5′end of MicA and GcvB respectively. For *phoP*, a PCR fragment corresponding to nts −36 to +169 of *phoP* mRNA preceded by two G residues was amplified from genomic DNA using oligonucleotides 5′T7phoP and 3′T7phoP. After purification, these PCR products were used in *in vitro* transcriptions reactions with the T7 RNA polymerase of Stratagene (for *phoP*) or the T7 Megascript kit from Ambion (for MicA and GcvB) following manufacturer's instructions. After phenol extraction and precipitation with ammonium acetate, RNA were purified using G-50 Microspin columns (GE Healthcare).

### Toeprinting Assays

Toeprinting assays were adapted from Hartz *et al.* [48] as follows. 0.5 pmol. of *phoP* transcript were incubated with 2 pmol. of phoP-Cy5-probe#1, an oligonucleotide complementary to nts 52 to 71 of *phoP* ORF and labeled with a Cy5 group at its 5′ end, in a buffer containing 10 mM Tris-acetate pH 7.4, 60 mM ammonium chloride and 6 mM ß-mercaptoethanol. When required, sRNAs were added to this mix at the desired concentrations. These mixtures were denatured by heating at 80°C for 3 minutes, followed by a rapid cooling in an ethanol/solid CO_2_ mix. They were then thawed on ice and magnesium was added at a final concentration of 10 mM. For experiment of Figure 7A, an additional incubation step at 37°C for 10 minutes was performed at this stage. 190 µM dNTPs and 2.5 µM of initiator tRNA^fMet^ were added together with 0.5 µM 30S subunits, and the mixtures were incubated at 37°C for 10 minutes. 1 unit of AMV RT (Finnzymes) was then added and cDNA synthesis was performed at 37°C for 20 minutes. Reactions were stopped by addition of formamide and EDTA, analyzed on a 6% sequencing gel together with sequencing reactions and RT stops were visualized using a typhoon fluorescent scanner set up for Cy5 detection.

### Structure Probing and Protections

1.25 pmol. of *phoP* transcript, mixed in water with 6.25 pmol. of sRNA when required, were denatured as above. After a slow thaw-out on ice, samples were incubated at 37°C for 10 minutes in order to allow sRNA and *phoP* message to pair. Samples were then diluted in a buffer containing 50 mM sodium cacodylate pH 7.5, 10 mM magnesium acetate and 50 mM ammonium chloride prior to DMS treatment, or in the same buffer except that sodium cacodylate is replaced by sodium borate pH 8.0 prior to CMCT or Kethoxal treatment. After a 10 minutes incubation at 25°C, 1 µg of *L. lactis* 23S rRNA was added, followed by 0.1 volume of DMS or kethoxal (stock solutions are 1/30 in ethanol or at 4 mg/ml in 20% ethanol respectively) and samples were incubated at 25°C for 5 minutes. For CMCT treatment, 0.1 volume of 100 mg/ml CMCT in the previous buffer containing sodium cacodylate was added and samples were incubated at 25°C for 10 minutes. Modified RNA were then precipitated with ammonium acetate (and 100 mM sodium borate pH 8.0 for samples treated with Kethoxal) and resuspended in water (or in 12.5 mM sodium borate pH 8.0 for samples treated with Kethoxal).

For reverse transcription, phoP-Cy5-probe#2, complementary to nts 87 to 106 of phoP ORF, was added to those samples at the final concentration of 1 µM. This was followed by the addition of 2 units of AMV RT (Finnzymes), together with 1 mM dNTPs and 4 mM DTT. cDNA synthesis and analysis was then performed as described above.

## Supporting Information

Figure S1 GcvBmutR1R3 does not control *phoP* expression either *in vivo* or *in vitro*, but it represses *phoPmutR3*, suggesting that GcvB R1 region is dispensable for *phoP* control. (A) The ß-galactosidase activity of strains carrying either a wt P_BAD_-*phoP-lacZ* fusion (MG1585) or its mutR3 derivative (strain MG1586) and deleted for *gcvB* was assessed upon overexpression of MicA, GcvB or their derivatives. (B) Toeprint experiments showing that GcvB and GcvBmutR1, but not GcvBmutR3 or GcvBmutR1R3, inhibit ribosome binding to *phoP* mRNA *in vitro*.
(EPS)

Figure S2 Conservation of GcvB and complementarities between GcvB and *phoP* in enterobacteriaceae. We focused on 20 species, each belonging to a family of the order of enterobacteriaceae. Using previous alignments (Sharma et al., 2007, Genes Dev, 21, 2804-17), we defined sequences conserved in enterobacteriaceae that are located close to the start and the end of gcvB sequences. Using these conserved sequences, we were able to characterize GcvB in 17 species each belonging to a different family. (S2A) Clustal alignment of GcvB in Enterobacteriaceae. The R3 region, as defined in the results, is one of the most conserved region of GcvB. Using Mfold, we looked at the pairing between the R3 region of all the selected species with the corresponding translation initiation region (TIR) of *phoP*. The alignments of Figure S2B show that good complementarities (5 or more consecutive nucleotides) can be found between R3 and the Shine-Dalgarno region of *phoP* in the cases of *Klebsiella pneumoniae* (5 consecutive nts) *Photorhabdus luminescens* (11 consecutive nts), *Proteus mirabilis* (6 consecutive nts), *Serratia proteamaculans* (5 consecutive nts), *Shigella flexneri* (8 consecutive nts), *Xenorhabdus bovienii* (14 consecutive nts), and *Rahnella* (5 consecutive nts). Since *phoP* is a target of GcvB in *E. coli* but does not seem to be in *Salmonella enterica*, one may conclude that either *E. coli* or *S. enterica* are an exception among enterobacteria. In fact our preliminary analysis suggests that neither of these bacteria are unique but that a subset of enterobacteria behave as *E. coli* and another as *S. enterica*. At any rate, only experimental results might tell us what these subsets are really composed of.
(PDF)

Figure S3 The different effect of GcvB and MicA is not due to transcripts initiating at P_2_ (A) and is independent of the promoter region of *phoPQ* (B). The ß-galactosidase activity of *phoP-lacZ* fusions driven by a P_tet_ promoter and whose 5′ end is expected to correspond to transcription starting at P_1_ (MG1511) or at P_2_ (MG1793) was assessed upon overexpression of MicA, GcvB and their derivatives. (B) Control of *ompT-lacZ* and *yneM-lacZ* by different sRNAs when *phoPQ* is expressed from a non native P_BAD_ promoter. Strains used in this experiment are MG1717 (*ompT-lacZ*; P_BAD_-*phoPQ*) and MG1718 (*yneM-lacZ*; P_BAD_-*phoPQ*). Material and methods for Figure S3: construction of strains was as follows. For strain MG1793, a PCR fragment carrying a P_tet_ promoter followed by nts −61 to +30 of *phoP* (relative to ATG) and nts +28 to +67 of *lacZ* ORF was amplified from genomic DNA of strain MG1655 using primers 5′Ptet-P2phoP (GATAGAGATTGACATCCCTATCAGTGATAGAGATACTGAGCACacccccataaccacataatcg) and 3′phoP-lacZ (Table S1). This PCR fragment was then recombined in strain MG1508 as described in the main text. Strains MG1715 and MG1718 were obtained by P1 transduction of CmR-P_BAD_-*phoPQ* (see main text) into strains MG1173 and KM194 respectively. ß-galactosidase activity was measured as decribed in the main text with cells grown in LB-Tetracyclin-IPTG (Figure S3A) or LB-Tetracyclin-IPTG-Arabinose 0.002% (Figure S3B).
(EPS)

Table S1 Oligonucleotides used in this study. Nts in upper cases correspond to homology regions for recombineering. Underlined nts indicate restriction sites used for cloning or T7 promoter sequence, and bold nts indicate mutations.
(PDF)

Table S2 ß–galactosidase activities. Shown are the average values of at least two independent experiments. Activities are expressed in Miller units (Mu), arbitrary units (au) or relative to the same strain transformed with the vector control, and whose activity is set up at 100% (%, experiment of Figure 1A and Figure S3B).
(PDF)
