# Supplementary material for: Post-Transcriptional Control of the Escherichia coli PhoQ-PhoP Two-Component System by Multiple sRNAs Involves a Novel Pairing Region of GcvB
Source: PLoS Genet. 2013 Jan 3;9(1):e1003156. doi: 10.1371/journal.pgen.1003156 (PMC3536696; doi:10.1371/journal.pgen.1003156)
Supplement: Figure S2 — Conservation of GcvB and complementarities between GcvB and phoP in enterobacteriaceae. We focused on 20 species, each belonging to a family of the order of enterobacteriaceae. Using previous alignments (Sharma et al., 2007, Genes Dev, 21, 2804-17), we defined sequences conserved in enterobacteriaceae that are located close to the start and the end of gcvB sequences. Using these conserved sequences, we were able to characterize GcvB in 17 species each belonging to a different family. (S2A) Clustal alignment of GcvB in Enterobacteriaceae. The R3 region, as defined in the results, is one of the most conserved region of GcvB. Using Mfold, we looked at the pairing between the R3 region of all the selected species with the corresponding translation initiation region (TIR) of phoP. The alignments of Figure S2B show that good complementarities (5 or more consecutive nucleotides) can be found between R3 and the Shine-Dalgarno region of phoP in the cases of Klebsiella pneumoniae (5 consecutive nts) Photorhabdus luminescens (11 consecutive nts), Proteus mirabilis (6 consecutive nts), Serratia proteamaculans (5 consecutive nts), Shigella flexneri (8 consecutive nts), Xenorhabdus bovienii (14 consecutive nts), and Rahnella (5 consecutive nts). Since phoP is a target of GcvB in E. coli but does not seem to be in Salmonella enterica, one may conclude that either E. coli or S. enterica are an exception among enterobacteria. In fact our preliminary analysis suggests that neither of these bacteria are unique but that a subset of enterobacteria behave as E. coli and another as S. enterica. At any rate, only experimental results might tell us what these subsets are really composed of. (PDF) [file pgen.1003156.s002.pdf]

S2A. Clustal alignment of GcvB in Enterobacteriaceae

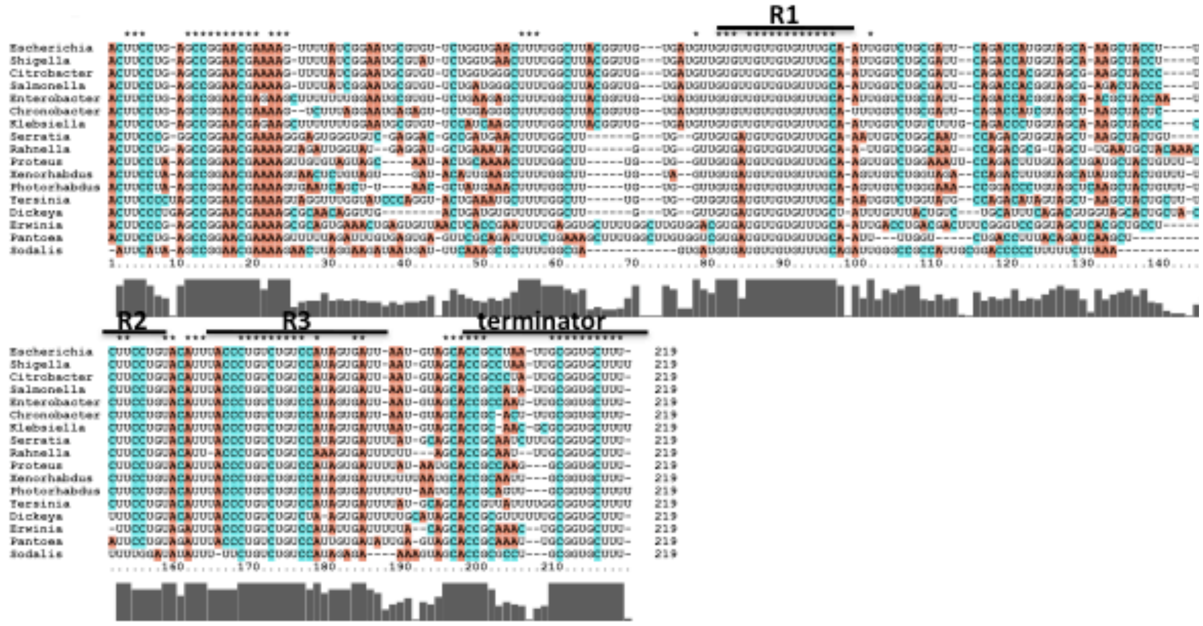

S2B. Complementarities between *gcvB* sRNAs and *phoP* mRNAs of several enterobacterial families.

The complementarities were found with the Mfold program using the default parameters and GcvB sequences in the R3 region and *phoP* sequences from -90 to +30 (+1 being the first nt of the translation initiation codon). In some cases, complementarities were lengthened by eye (in blue). The Shine-Dalgarno sequence and the initiation codon are in red. The maximum continuous complementarity length is given under each sequence.

E. coli K12 MG1655
phoP 5'UUUUAAUAAUUA-----AGACAGGGAGAAAUAAAAAUGCGCGUACUGG3'
gcvB 3'GUAAUUAGUGAUACCUGUCUGUCCCAUUUAC5'

Maximum complementarity length : 8 nt

Klebsiella pneumoniae 342
phoP 5'UUA-----CU-AUCAACACAGGAAGGGAGAACAGUCAUGCGCGUACUCG3'
gcvB 3'UAAUUUAGA-UACC--UGUCUGUCCCAUUUAC5'

Maximum complementarity length : 5 nt

Photorhabdus luminescens TT01
phoP 5'AAUAACUGCC-UGCUGGAGAGGAAUUAUUCCAUGCGGAUAUUGA3'
gcvB 3'TUUUCGUGGCGUUGACGCCAC-GUAAUUUUUUAGUGAUACCUGUCUGUCCCAUU5'

Maximum complementarity length : 11 nt

Proteus mirabilis HI4320
phoP 5'UGAUUUUUACUGAUGUAC--ACAAGGUGGAAUAUGCGGAUCUUAA3'
gcvB 3'UUUUAGUG-AUACCUGUCUGUCCCAUUUAC5'

Maximum complementarity length : 6 nt

Serratia proteamaculans 568

phoP 5'UGUUCACCCGCAGAUUUACAGGAGUCCGCCAUGCGAGUACUGG3'

gcvB 3'UUUUAGU-GAUACCUGUCUGUCC-CAUUUAC5'

Maximum complementarity length : 5 nt

Shigella flexneri

phoP 5'UUA--CACUAUUUUAAUAAUUAAGACAGGGAGAAAUAAAAAUGCGCGUACUGG

gcvB 3'GUAAUUAGUGAUA-------CCUGUCUGUCCCAUUUAC

Maximum complementarity length : 8 nt

Xenorhabdus bovienii SS-2004

phoP 5'GUAAACGUUACUGAUUAGUAUAAGGAAUCAUUAUGCGUAUACUCA

gcvB 3'AAUUUUUUUAGUGAUACCUGUCUGUCCCAUU

Maximum complementarity length : 14 nt

Rahnella sp. Y9602

phoP 5'TUUUUCGCGCUGUCAGGAUAAGG-AAUCACCAUGCGAAUUUUAG

gcvB 3'UUAGUGAAACCUGUCUGUCCCAUUACAU5'

Maximum complementarity length : 5 nt
