# Supplementary material for: Post-Transcriptional Control of the Escherichia coli PhoQ-PhoP Two-Component System by Multiple sRNAs Involves a Novel Pairing Region of GcvB
Source: PLoS Genet. 2013 Jan 3;9(1):e1003156. doi: 10.1371/journal.pgen.1003156 (PMC3536696; doi:10.1371/journal.pgen.1003156)
Supplement: Table S1 — Oligonucleotides used in this study. Nts in upper cases correspond to homology regions for recombineering. Underlined nts indicate restriction sites used for cloning or T7 promoter sequence, and bold nts indicate mutations. (PDF) [file pgen.1003156.s004.pdf]

Table S1. Oligonucleotides used in this study

Primer	5’-3’ sequence	Use
**Strains construction**		
ΔgcvB ::tetfor	GTCCGTTGAGCTTCTACCAGCAAATACCTATAGTGGCGGCACctagacatcattaattccta	Construction *ΔgcvB ::tet* allele
ΔgcvB ::tetrev	CGATCGCAAGGTAAAAAAAAGCACCGCAATTAGGCGGTGCTgaagctaaatcttctttatcg	
ΔgcvB ::kanfor	GTCCGTTGAGCTTCTACCAGCAAATACCTATAGTGGCGGCACaaagccacgttgtgtctcaa	Construction *ΔgcvB ::kan* allele
ΔgcvB ::kanrev	CGATCGCAAGGTAAAAAAAAGCACCGCAATTAGGCGGTGCTttagaaaaactcatcgagc	
5’Ptet-phoP	GATAGAGATTGACATCCCTATCAGTGATAGAGATACTGAGCACacactattttaataattaag acaggg	Construction Ptet-*phoP-lacZ* fusion
3’phoP-lacZ	TAACGCCAGGGTTTTCCCAGTCACGACGTTGTAAAACGACcgcattgtcttcaacaaccag	
phoPmutR3for	ACCTGACGCTTTTTATCGCAACTCTCTACTGTTTCTCCATacactattttaataattaa**ctgtc**gga gaaataaaaatgcgcgtactgg	Construction P_{BAD}-*phoPmutR3-lacZ* fusion
5’P1mgtA	GCGAAGCGGCATGCATTTACGTTGACACCATCGAATGGCGCgtgcattcagcaatgggtaaa gtc	Construction P1-*mgtA-lacZ* fusion
3’mgtA-lacZ	GTAACGCCAGGGTTTTCCCAGTCACGACGTTGTAAAACGACaatgagccgggtaaaatttctt	
5'LivJ-lac	ACCTGACGCTTTTTATCGCAACTCTCTACTGTTTCTCCATagagtatgctgctaaagcacg	Construction P_{BAD}-*livJ-lacZ* fusion
3'LivJ-lac	TAACGCCAGGGTTTTCCCAGTCACGACGTTGTAAAACGACtatgttcatcctgagaatccc	
5’Cm-P_{BAD}-phoPQ	AAGATTATCCGCTTTTTATTTTTTCACTTTACCTCCCCTCCgtgtaggctggagctgct	Construction CmR-P_{BAD}-*phoPQ* allele
3’Cm-P_{BAD}-phoPQ	GCATTTTTATTTCTCCCTGTCTTAATTATTAAAATAGTGTatggagaaacagtagagagt	
**Plasmid construction**		
pBR-for	ggaaaacgttcttcggggcg	
5’Hind-MicA-tet	cgtacaagcttatcgatgataagctgtcaaacatgagaattccacgcctgacagaaaagaaaaaggc	
GcvBmutR1for	ggcttacggttgtgatgttg**acaaca**tgtgtttgcaattggtctgcgattc	
GcvBmutR1rev	gaatcgcagaccaattgcaaacaca**tgttgt**caacatcacaaccgtaagcc	

GcvBmutR3for	ttttcacttcctgtacatttacc**gacag**tgtccatagtgattaatgtagc	
GcvBmutR3rev	gctacattaatcactatggaca**ctgtc**ggtaaatgtacaggaagtgaaaa	
**Probes for Northern-Blot**		
MgrR-probe	5’Bio-cagtaaaccggcggtgaatgcttgcatggatagatttgtg	
ompT-probe	5’Bio-gccgatatcatctctgaatccctcctcagaactgtagatatagg	
SsrA-probe	5’Bio-cgccactaacaaactagcctgattaagttttaacgcttca	
GcvB-probe	5’Bio-cacaaccgtaagccaaaagttcaccagaacacgcattccg	
phoP-probe	5’Bio-gcatcttctgcgtcatcgacctgatgaccagcatcctgaatc	
**In vitro experiments**		
5'T7MicA	taatacgactcactataggaaagacgcgcatttg	
5’T7MicAmut	taatacgactcactataggaaaga**gcgc**catttg	
3’T7MicA	aaaaggccactcgtgagtgg	
5’T7GcvB	taatacgactcactataggacttcctgagccggaacg	
3’T7GcvB	ggtaaaaaaaagcaccgcaattaggcgg	
5’T7phoP	taatacgactcactataggacactattttaataattaagacaggg	
3’T7phoP	cgtctggcaatccgagatc	
phoP-Cy5-probe#1	5'Cy5-ccagcatcctgaatctgaac	
phoP-Cy5-probe#2	5'Cy5-cttctttggcatcttctgcg	

Nts in upper cases correspond to homology regions for recombineering
Underlined nts indicate restriction sites used for cloning or T7 promoter sequence, and bold nts mutations
