# Supplementary material for: Post-Transcriptional Control of the Escherichia coli PhoQ-PhoP Two-Component System by Multiple sRNAs Involves a Novel Pairing Region of GcvB
Source: PLoS Genet. 2013 Jan 3;9(1):e1003156. doi: 10.1371/journal.pgen.1003156 (PMC3536696; doi:10.1371/journal.pgen.1003156)
Supplement: Table S2 — ß–galactosidase activities. Shown are the average values of at least two independent experiments. Activities are expressed in Miller units (Mu), arbitrary units (au) or relative to the same strain transformed with the vector control, and whose activity is set up at 100% (%, experiment of Figure 1A and Figure S3B). (PDF) [file pgen.1003156.s005.pdf]

Table S2. β-galactosidase activities

Activities are expressed in Miller units (Mu), arbitrary units (au) or relative to the same strain transformed with the vector control, and whose activity is set up at 100% (%, experiment of Fig. 1A and S3B)

Strain tested	Average β-galactosidase activity	Standard deviation	Figure
MG1425/pSgrS	217,9 %	23,7	1A
MG1425/pRydC	204,2 %	40,0	1A
MG1425/pRybC	188,7 %	14,0	1A
MG1425/pRprA	185,1 %	25,7	1A
MG1425/pRydD	173,0 %	19,4	1A
MG1425/pRyhB	171,5 %	32,0	1A
MG1425/pMicC	161,3 %	5,47	1A
MG1425/pRyhA	159,7 %	23,4	1A
MG1425/pOxyS	152,9 %	24,9	1A
MG1425/pRybB	145,0 %	5,67	1A
MG1425/pOmrA	142,9 %	0,76	1A
MG1425/pOmrB	137,7 %	13,0	1A
MG1425/pRseX	121,1 %	5,18	1A
MG1425/pMicF	115,8 %	11,5	1A
MG1425/pGlmZ	106,2 %	3,74	1A
MG1425/pBRplac	100		1A
MG1425/pSpk	97,4 %	0,83	1A
MG1425/pGadY	93,1 %	1,88	1A
MG1425/pCyaR	92,7 %	9,95	1A
MG1425/pGlmY	91,8 %	7,21	1A
MG1425/pDicF	87,8 %	9,48	1A
MG1425/pRyeB	84,6 %	7,94	1A
MG1425/pDsrA	69,2 %	9,66	1A
MG1425/pSpot42	59,8 %	0,56	1A
MG1425/pMicA	33,2 %	5,24	1A
MG1425/pGcvB	23,2 %	1,57	1A
MG1511/pBRplac	1760 Mu	53,0	1B
MG1511/pGcvB	493,2 Mu	20,5	1B
MG1511/pSgrS	1813 Mu	126,9	1B
MG1511	11,3 au	1,06	1C
MG1521	21,5 au	1,32	1C
MG1452/pBRplac	1010 Mu	229.4	2A
MG1452/pMicA	316.4 Mu	45.8	2A
MG1452/pGcvB	236.8 Mu	50.2	2A
MG1425	596.8 Mu	36	2B
MG1768	1038 Mu	249	2B
MG1430	516.2 Mu	49.2	2B
MG1709	1163 Mu	40.2	2B
MG1428	397.3 Mu	57.7	2B

MG1769	880.2 Mu	94.6	2B
MG1585/pBRplac	13.99 au	1.34	3C
MG1585/pGcvB	2.10 au	0.63	3C
MG1585/pGcvBmutR1	3.04 au	0.41	3C
MG1585/pGcvBmutR3	29.0 au	2.01	3C
MG1585/pMicA	3.30 au	0.33	3C
MG1585/pMicAmut	10.7 au	0.95	3C
MG1586/pBRplac	20.0 au	1.80	3C
MG1586/pGcvB	29.0 au	0.84	3C
MG1586/pGcvBmutR1	22.5 au	2.01	3C
MG1586/pGcvBmutR3	2.38 au	1.37	3C
MG1586/pMicA	1.46 au	1.38	3C
MG1586/pMicAmut	19.4 au	1.40	3C
AC0067/pBRplac	19.5 Mu	0.44	3D
AC0067/pGcvB	7.57 Mu	0.89	3D
AC0067/pGcvBmutR1	14.1 Mu	1.15	3D
KM112/pBRplac	7509 Mu	484	6A
KM112/pMicA	2991 Mu	55	6A
KM112/pMicAmut	7992 Mu	456	6A
KM112/pGcvB	7866 Mu	393	6A
KM112/pGcvBmutR1	3910 Mu	117	6A
KM112/pGcvBmutR3	7228 Mu	353	6A
MG1173/pBRplac	296.1 Mu	10.8	6A
MG1173/pMicA	108.2 Mu	6.1	6A
MG1173/pMicAmut	300.7 Mu	13.0	6A
MG1173/pGcvB	324.0 Mu	17.9	6A
MG1173/pGcvBmutR1	122.9 Mu	5.01	6A
MG1173/pGcvBmutR3	387.5 Mu	56.4	6A
MG1528/pBRplac	105.6 Mu	3.1	6A
MG1528/pMicA	40.1 Mu	1.3	6A
MG1528/pMicAmut	112.4 Mu	6.4	6A
MG1528/pGcvB	169.9 Mu	29.5	6A
MG1528/pGcvBmutR1	59.2 Mu	5.7	6A
MG1528/pGcvBmutR3	93.6 Mu	2.9	6A
KM194/pBRplac	239.1 Mu	1.19	6A
KM194/pMicA	82.5 Mu	4.47	6A
KM194/pMicAmut	215.2 Mu	6.80	6A
KM194/pGcvB	495.9 Mu	24.7	6A
KM194/pGcvBmutR1	114.2 Mu	3.22	6A
KM194/pGcvBmutR3	397.7 Mu	38.1	6A
MG1585/pBRplac	22.8 au	0.02	S1A
MG1585/pMicA	7.0 au	1.10	S1A
MG1585/pMicAmut	25.2 au	4.49	S1A
MG1585/pGcvB	3.79 au	0.86	S1A
MG1585/pGcvBmutR1	4.3 au	0.51	S1A
MG1585/pGcvBmutR1R3	35.6 au	6.05	S1A
MG1586/pBRplac	37.8 au	0.88	S1A
MG1586/pMicA	5.92 au	0.83	S1A

MG1586/pMicAmut	45.2 au	5.61	S1A
MG1586/pGcvB	44.1 au	2.81	S1A
MG1586/pGcvBmutR1	45.5 au	9.40	S1A
MG1586/pGcvBmutR1R3	4.05 au	0.43	S1A
MG1511/pBRplac	1012 Mu	76.2	S3A
MG1511/pMicA	342.9 Mu	13.63	S3A
MG1511/pMicAmut	1144 Mu	54.2	S3A
MG1511/pGcvB	382.6 Mu	71.4	S3A
MG1511/pGcvBmutR1	279.0 Mu	20.6	S3A
MG1511/pGcvBmutR3	1163 Mu	186.1	S3A
MG1793/pBRplac	562.8 Mu	54.4	S3A
MG1793/pMicA	262.5 Mu	8.3	S3A
MG1793/pMicAmut	546.0 Mu	6.8	S3A
MG1793/pGcvB	229.6 Mu	7.5	S3A
MG1793/pGcvBmutR1	188.6 Mu	13.1	S3A
MG1793/pGcvBmutR3	654.4 Mu	92.0	S3A
MG1715/pBRplac	100		S3B
MG1715/pMicA	49.9%	10.6	S3B
MG1715/pMicAmut	107.2%	25.3	S3B
MG1715/pGcvB	76.6%	2.0	S3B
MG1715/pGcvBmutR1	35.8%	4.6	S3B
MG1715/pGcvBmutR3	126.1%	49.3	S3B
MG1718/pBRplac	100		S3B
MG1718/pGcvB	364.9%	40.4	S3B
